# Peripheral neuropathy associated with monomethyl auristatin E-based antibody-drug conjugates

**DOI:** 10.1016/j.isci.2023.107778

**Published:** 2023-08-29

**Authors:** Zhiwen Fu, Chen Gao, Tingting Wu, Lulu Wang, Shijun Li, Yu Zhang, Chen Shi

**Affiliations:** 1 Department of Pharmacy, Union Hospital, Tongji Medical College, Huazhong University of Science and Technology, Wuhan 430000, China; 2 Hubei Province Clinical Research Center for Precision Medicine for Critical Illness, Wuhan 430000, China

**Keywords:** Health sciences, Medicine, Toxicology, Oncology, Cancer systems biology, Cancer

## Abstract

Since the successful approval of gemtuzumab ozogamicin, antibody-drug conjugates (ADCs) have emerged as a pivotal category of targeted therapies for cancer. Among these ADCs, the use of monomethyl auristatin E (MMAE) as a payload is prevalent in the development of ADC drugs, which has significantly improved overall therapeutic efficacy against various malignancies. However, increasing clinical observations have raised concerns regarding the potential nervous system toxicity associated with MMAE-based ADCs. Specifically, a higher incidence of peripheral neuropathy has been reported in ADCs incorporating MMAE as payloads. Considering the increasing global use of MMAE-based ADCs, it is imperative to provide an inclusive overview of diagnostic and management strategies for this adverse event. In this review, we examine current information and what future research directions are required to better understand and manage this type of clinical challenge.

## Graphical abstract

**Figure:**
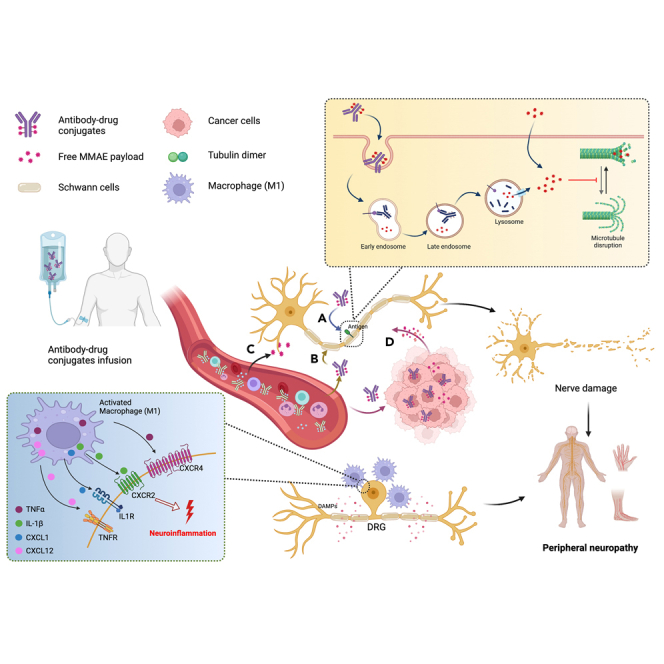

## Introduction

The approval of gemtuzumab ozogamicin in 2000 marked a significant milestone in the development of antibody-drug conjugates (ADCs) as a crucial class of targeted therapies for cancer.^1^ An ADC comprises a tumor-targeting monoclonal antibody (mAb) chemically conjugated to a highly cytotoxic payload via a linker, facilitating precise delivery of the cytotoxic agent into cancer cells. Acting as a biological missile, the cytotoxic payload of ADC is the warhead that exerts cytotoxicity to kill cancer cells. The use of monomethyl auristatin E (MMAE) as a payload is prevalent in the development of ADC drugs, with five out of the fifteen approved ADCs utilizing this agent (Table 1).^2^ Upon recognition and binding of target antigens expressed on the surface of cancer cell, the ADC is internalized via endocytosis, and the payload is subsequently released through proteolytic/acidic cleavage to achieve accurate killing. Through its interaction with tubulin, MMAE inhibits the formation of microtubules and disrupts the assembly of the mitotic spindle, resulting in the arrest of tumor cells in the M phase of the cell cycle.^3^

**Table 1:** Summary of antibody–drug conjugates with MMAE payloads that were approved for market worldwide, as of March, 2023

ADC drugs	Trade names	Target antigens	Linkers	Payloads	Average DAR	Approved countries	Approved date	Approved indications
Brentuximab vedotin	Adcetris	CD30	mc-VC-PABC	MMAE	4	FDA/EMA//NMPA	2011/8/19	R/R CD30 positive HL and systemic ALCL; in combination with chemotherapy including the treatment of certain types of PTCL and previously untreated stage III or IV cHL.
Polatuzumab vedotin	Polivy	CD79B	mc-VC-PABC	MMAE	3.5	FDA/EMA	2019/6/10	in combination with bendamustine plus rituximab for the treatment of patients with R/R DLBCL, who have received at least two prior therapies.
Enfortumab vedotin	Padcev	Nectin-4	mc-VC-PABC	MMAE	3.8	FDA	2019/12/18	locally advanced or metastatic urothelial cancer who have previously received platinum chemotherapy and a PD-L1/PD-1 inhibitor
Disitamab vedotin	Aidixi	HER2	mc-VC-PABC	MMAE	4	NMPA	2021/6/8	patients with locally advanced or metastatic gastric cancer (including gastroesophageal junction adenocarcinoma) who have received at least 2 types of systemic chemotherapy
Tisotumab vedotin	Tivdak	TF	mc-VC-PABC	MMAE	4	FDA	2021/9/20	adult patients with recurrent or metastatic cervical cancer with disease progression on or after chemotherapy, which is the first and only approved TF-directed ADC therapy

FDA, US Food and Drug Administration; EMA, European Medicines Agency; NMPA, National Medical Products Administration of China; DAR, Drug-to-Antibody Ratio; R/R, relapsed or refractory; AML, acute myeloid leukemia; mc-VC-PABC, maleimidocaproyl-valine-citrulline-*p*-aminobenzoyloxycarbonyl; MMAE, monomethyl auristatin E; HL, Hodgkin lymphoma; ALCL, anaplastic large cell lymphoma; HER2, human epidermal growth factor receptor 2; cHL, classical Hodgkin lymphoma; PTCL, peripheral T-cell lymphomas. MM, multiple myeloma. DLBCL, diffuse large B-cell lymphoma; TF, tissue factor.

The MMAE-based ADCs have shown remarkable efficacy in improving overall therapeutic outcomes against various cancers. For instance, brentuximab vedotin treatment in CD30-positive Hodgkin lymphoma and systemic anaplastic large-cell lymphoma patients resulted in overall objective response rates (ORR) of 73% and 86%, respectively.^4^^,^^5^ Additionally, polatuzumab vedotin combined with bendamustine and rituximab significantly increased the complete response rate from 18% to 40% in patients with relapsed or refractory diffuse large B-cell lymphoma.^6^ Concerning the solid tumors, enfortumab vedotin has demonstrated remarkable efficacy in the treatment of locally advanced or metastatic urothelial cancer. When compared to other chemotherapy regimens, it could induce a significant extension in median overall survival (12.9 vs. 9.0 months) and median progression-free survival (5.6 vs. 3.7 months).^7^^,^^8^ In addition, tisotumab vedotin exhibited promise in treating patients suffering from recurrent or metastatic cervical cancer. It achieved an objective response rate of 24% and a median response duration of 8.3 months.^9^

However, increasing clinical observations have raised concerns regarding the potential toxicity to nervous system caused by MMAE-based ADCs, predominantly manifesting as peripheral neuropathy.^10^ A recent meta-analysis of payload-related clinical toxicity in ADCs revealed that ADCs containing MMAE payloads have a higher risk of causing peripheral neuropathy.^11^ Peripheral neuropathy encompasses a range of peripheral nervous system disorders, including weakness, numbness, and pain.^12^ The development of peripheral neuropathy may result in prolonged infusion times, or dose reduction, which negatively impact both treatment efficacy and patient quality of life. In severe cases, peripheral neuropathy may even threaten the patient’s life.^13^

Considering the increasing global use of MMAE-based ADCs, in this review, we provide an inclusive overview of peripheral neuropathy induced by MMAE-based ADCs. Through comprehensively addressing the clinical aspects, underlying mechanisms, and management strategies, our work aims to facilitate a better understanding of this adverse effect and inform future research efforts to enhance the safety and efficacy of ADC therapy.

## Epidemiology

**Figure 1:**
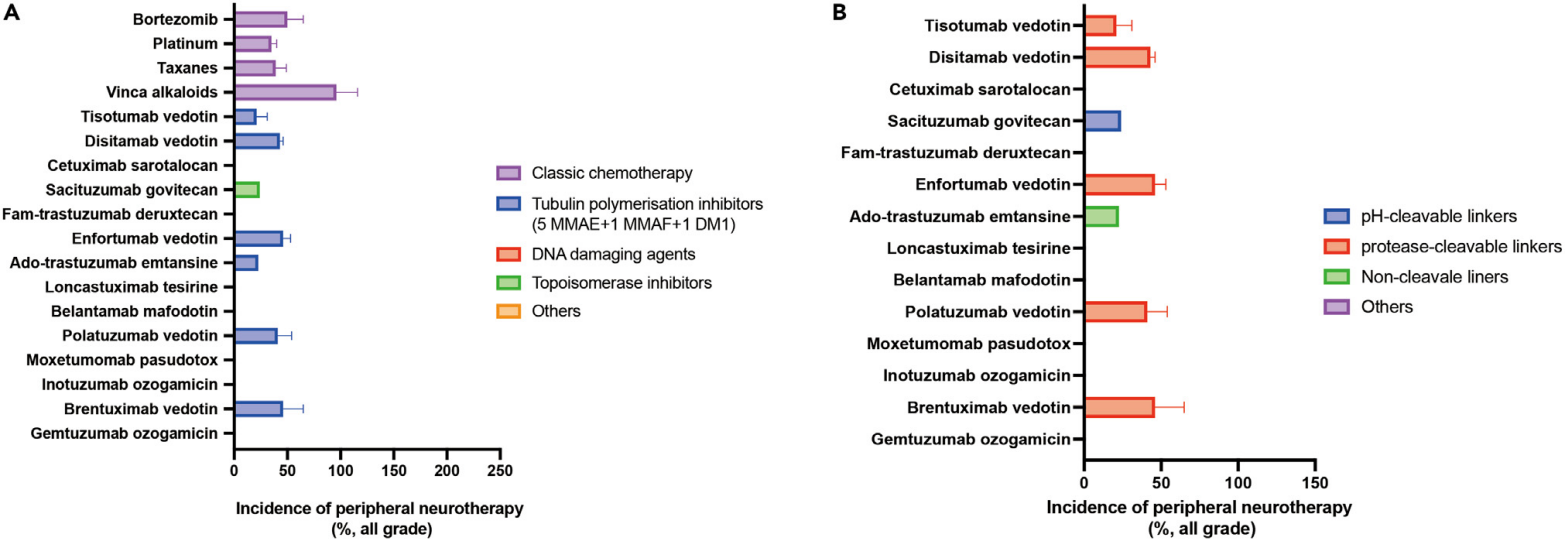
The risk differece of peripheral neuropathy among approved antibody-drug conjugates The mean incidences of peripheral neuropathy of any grade by payloads (A) and linkers (B).

Peripheral neuropathy is a commonly observed adverse effect of microtubule-targeting agents in clinical trials, with varying frequencies and levels of severity.^14^ For example, the representative chemotherapy agents including bortezomib, platinum, taxanes, and vinca alkaloids had a higher incidences of peripheral neuropathy (all-grade, varying between 15% and 90%).^15^ As for approved ADC drugs, those with tubulin polymerization inhibitor, in particular with MMAE, as the payload exhibited the significant observation of peripheral neuropathy^1^ (Figure 1). A systematic review of 4,367 cancer patients treated with microtubule-stabilizing agents revealed that the overall incidence of severe peripheral neuropathy (grade 3 or 4) was as high as 30%.^16^ When restricting the analysis to ADCs containing MMAE payloads, the incidence of grade 3 or 4 toxicity for peripheral neuropathy was 6.5% (90% CI, 4.4%–9.4%).^11^ The rates of peripheral neuropathy reported in clinical trials investigating MMAE-based ADCs are summarized in Table 2.

**Table 2:** The rates of peripheral neuropathy reported in clinical trials investigating MMAE-based ADCs

Drugs	Indications	No. of patients	Incidence (%)		Reference
			Any-grade	Grade 3 or 4	
Brentuximab vedotin	Relapsed/refractory CD30-positive HL and ALCL	45	22%	0%	Anas Younes (2010)^17^
	Relapsed/refractory systemic ALCL	58	41%	12%	Barbara Pro (2012)^18^
	Relapsed/refractory CD30-positive NHL	35	–	9%	Steven Horwitz (2014)^19^
	CD30-positive B-cell lymphomas, including DLBCL and other B-cell lymph	68	26.5%	2.9%	Eric D. Jacobsen (2015)^20^
	CD30-positive mycosis fungoides or primary cutaneous ALCL	66	67％	9%	H Miles Prince (2017)^21^
	CD30-positive PTCL^a^	223	52%	4%	Steven Horwitz (2019)^22^
	Stage III or IV classic HL^a^	662	66%	1.6%	Stephen M. Ansell (2022)^23^
Polatuzumab vedotin	Relapsed/refractory B-cell NHL and CLL	45	27%	9%	Maria Corinna A Palanca-Wessels (2015)^24^
	Relapsed/refractory DLBCL^a^	39	43.6%	0%	Laurie H. Sehn (2020)^25^
	CD20-positive DLBCL^a^	435	52.9%	1.6%	H. Tilly (2022)^26^
Enfortumab vedotin	Locally advanced or metastatic UC who had previously received platinum-based chemotherapy and previously treated with PD-1 or PD-L1 inhibitors.	296	46.3%	5.1%	Thomas Powles (2021)^27^
	Locally advanced or metastatic UC previously treated with PD-1 or PD-L1 inhibitors	125	40%	2%	Evan Y Yu (2021): Cohort 1^28^
	Locally advanced or metastatic UC previously treated with PD-1 or PD-L1 inhibitors	89	54%	7.9%	Evan Y Yu (2021): Cohort 2^28^
Disitamab vedotin	HER2-positive locally advanced or mUC who previously failed at least one line of systemic chemotherapy	43	46.3%	2.3%	Xinan Sheng (2021)^29^
	HER2-positive locally advanced or metastatic gastric or gastroesophageal junction cancer who were under at least second-line therapy	125^b^	40%	3.2%	Zhi Peng (2021)^30^
Tisotumab vedotin	Relapsed, advanced, or metastatic cancer of the ovary, cervix, endometrium, bladder, prostate, esophagus, HNSCC or NSCLC	147	21%	1%	Johann S de Bono (2019)^31^
	Recurrent or metastatic cervical cancer	55	36%	4%	David S Hong (2020)^32^
	Recurrent or metastatic cervical cancer who had received no more than two prior systemic regimens	101	8%	2%	Robert L Coleman (2021)^33^
	Japanese patients with advanced solid malignancies	17	17.6%	0%	Kan Yonemori (2022)^34^

ALCL, anaplastic large-cell lymphoma; HL, Hodgkin’s lymphoma; PTCL, peripheral T cell cell lymphomas; NHL, non-Hodgkin lymphomas; CLL, chronic lymphocytic leukemia; DLBCL, diffuse large B-cell lymphoma; UC, urothelial carcinoma; NSCLC, non-small-cell lung cancer; HNSCC, head and neck squamous cell carcinoma.

a Combination with chemotherapy.

b Described as hypoesthesia.

The incidence of peripheral neuropathy of any grade in patients treated with brentuximab vedotin was reported to be between 36 and 69 percent, with approximately 4–14 percent of patients experiencing severe peripheral neuropathy.^35^^,^^36^^,^^37^^,^^38^ A similar frequency was reported for patients treated with enfortumab vedotin. In the EV-201 trial, treatment-related peripheral neuropathy was observed in 54% (48/89) of patients treated with enfortumab vedotin, with a median onset of 2.4 months after the initiation of treatment.^39^ Most cases (85%) of peripheral neuropathy were grade 1 or 2.^39^ In the expanded set of 296 patients treated with enfortumab vedotin in the EV-301 trial, treatment-related peripheral neuropathy, predominantly manifested as sensory events, was observed in 46.3% of patients, with 3.7% of the events being grade 3 peripheral neuropathy.^40^ Polatuzumab vedotin can also cause predominantly sensory peripheral neuropathy, which is typically mild (42% grade 1/2 and 9% grade 3/4).^41^ In addition, the incidence of peripheral neuropathy in the combination of polatuzumab vedotin with bendamustine and rituximab appeared similar to that of polatuzumab vedotin monotherapy, with 43.6% of patients experiencing any-grade peripheral neuropathy.^25^^,^^26^

Treatment with tisotumab vedotin was also associated with peripheral neuropathy. In the innovaTV-204 study, peripheral neuropathy treatment-related adverse events occurred in 33% of patients, with 26% having grade one or two events, and 7% having grade 3 events.^42^ Adverse events in this class have included peripheral neuropathy (10% of patients; 2% with grade 3), peripheral sensory neuropathy (9%; 2% with grade 3), and peripheral sensorimotor neuropathy (5%; 2% with grade 3).^42^ More recently, results from the innovaTV-206 trial showed that 17.6% experienced peripheral neuropathy in Japanese patients with recurrent/metastatic cervical cancer, which was consistent with the rates observed in the innovaTV-204 study.^34^ In addition, nervous system toxicities were observed and commonly reported as hypoesthesia (60.5%), pruritus (16.3%), and peripheral sensory neuropathy (14.0%) in the phase II study of disitamab vedotin.^43^ Nonetheless, peripheral sensory neuropathy induced by disitamab vedotin was manageable, as grade 3 toxicities were rare (2.3%).^43^

In addition to the specific risk factors identified in MMAE-based ADC clinical trials, it is worth noting that there are also general risk factors, previously observed with other anticancer agents, such as age, comorbidities, dose level (single dose and cumulative dose), infusion time, treatment duration, lifestyle factors (e.g., smoking, alcohol), and prior treatments with the other anticancer agents, which may increase the risk of peripheral neuropathy.^44^^,^^45^

## Pathophysiological mechanisms

Drug-induced peripheral neuropathy is a common form of nervous system toxicity, with chemotherapy agents being the primary cause. Chemotherapy drugs known to cause peripheral neuropathy potentially include platinum-based compounds (cisplatin, carboplatin, and oxaliplatin),^46^ vinca alkaloids (vincristine),^47^ and taxanes (paclitaxel and docetaxel),^48^ proteasome or angiogenic inhibitors (bortezomib and thalidomide),^49^ and MMAE-based ADCs.^50^ Pathologically, peripheral neuropathy is attributed to structural or functional damage to the peripheral nerves, typically causing weakness, numbness, and pain in the extremities. As a neuropathic pain, various mechanisms have been revealed to be associated with the development of peripheral neuropathy, such as glial cell activation, mitochondrial dysfunction, oxidative stress, ion channel changes, and neuroinflammation activation.^12^^,^^51^ Under this background, the specific mechanism of peripheral neuropathy caused by MMAE-based ADCs remains to be fully understood, despite the most likely involvement of carried MMAE payload.

**Figure 2:**
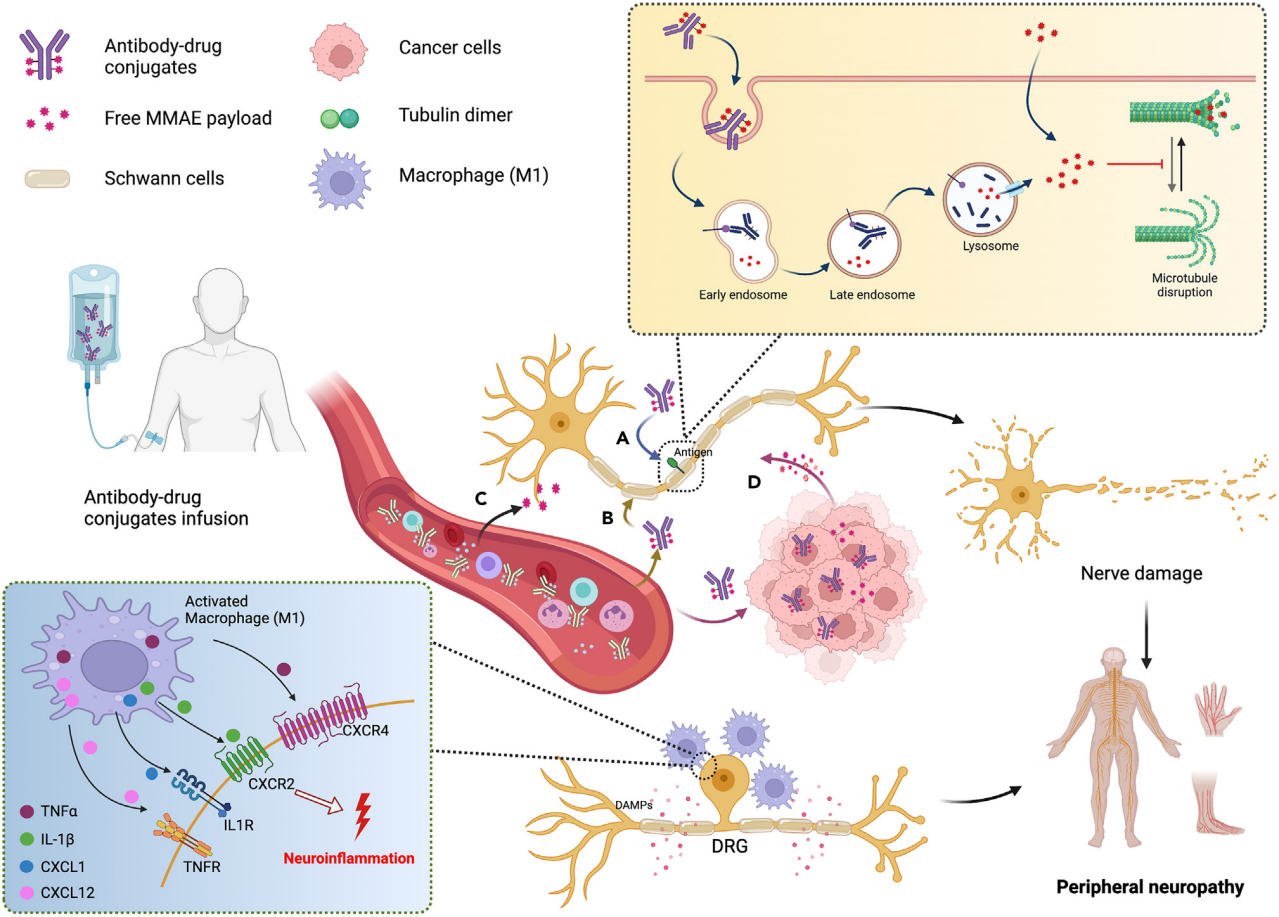
Possible mechanisms underlying peripheral neuropathy associated with MMAE-based antibody-drug conjugates (ADCs) (A) target-dependent uptake of the ADCs. (B) the non-specific uptake of ADCs by healthy cells. (C) the premature MMAE release during circulation. (D) the bystander effects of MMAE-based ADCs. Once the MMAE is taken up in the peripheral nerve cells through the direct method (free MMAE) or the indirect method (MMAE-based ADC uptake to release MMAE), MMAE binds to abundant binding sites along the length of microtubules and disrupts the microtubules network, resulting in the onset of peripheral neuropath. In addition, in cases where nerve injury is caused by microtubule-targeting drugs, Schwann cells become activated and produce damage-associated molecular patterns (DAMPs), which in turn attract circulating leukocytes, such as M1-like macrophages, to the site of injury. It promotes the release of various pro-inflammatory cytokines, including IL-1β, TNFα, CXCL1, CXCL12, *etc*. These cytokines trigger neuroinflammation and convey nociceptive information to DRG neurons, leading to the perception of pain. The Figures were created with BioRender.com.

Based on the properties of MMAE-based ADCs, the induced peripheral neuropathy may be theoretically attributed to: (1) the binding of ADCs to target antigens expressed in peripheral nerve cells resulting in the target-dependent uptake of the ADCs; (2) the non-specific uptake of ADCs by peripheral nerve cells or immune cells; (3) the bystander effects of MMAE-based ADCs; (4) the premature MMAE release during circulation due to unexpected cleavage (Figure 2). The direct and indirect uptake of MMAE by peripheral nerves causes the inhibition of proliferation, mitosis, and disruption microtubule network, thereby blocking microtubule-dependent axonal transport and causing severe peripheral neuropathy.^52^ Although the neurotoxicity underlying the available MMAE-based ADCs exhibits a target-independent mechanism,^10^^,^^53^ there is also a possibility to causes the direct uptake of ADC drugs when the same antigens that some ADC drugs target are present in the normal peripheral nerves cells and tissues. For the better advancement of next generations of ADCs, more effort should be rightfully allotted to the identification of tumor-specific targets which avoids the exposure of cytotoxic payloads in the healthy cells.

**Figure 3:**
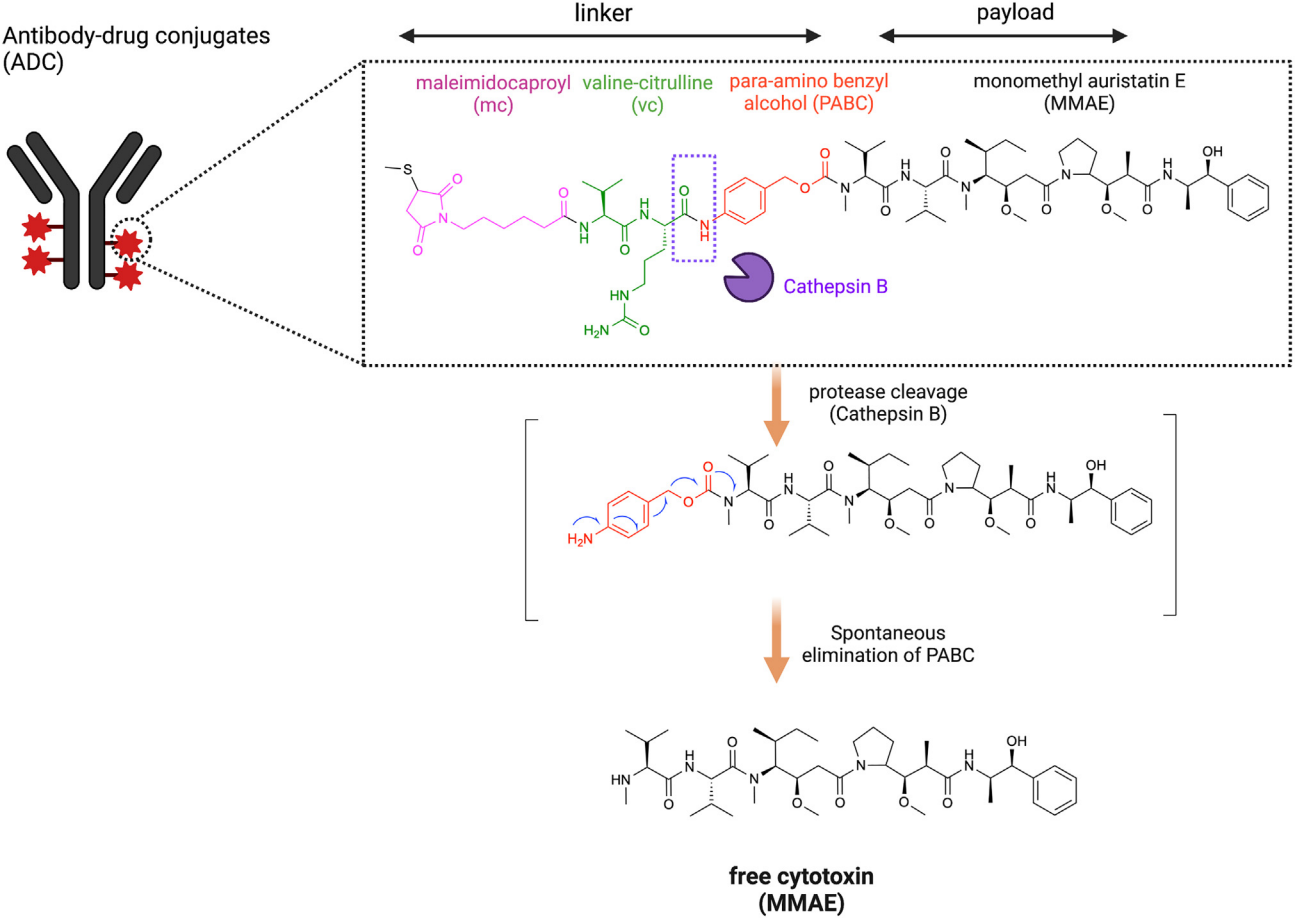
Chemical structure of MMAE-based antibody-drug conjugates (ADCs) The mc-vc-PABC cleavable linker is employed for the conjugation between payload and antibody, which comprised of a thiol-reactive maleimidocaproyl (mc) group, a valine-citrulline (vc) dipeptide, and a para-amino benzyloxycarbonyl (PABC) spacer. Vedotin, the mc-vc-PABC-MMAE drug/linker complex, undergoes the protease cleavage of valine-citrulline dipeptide linker by cathepsin B followed spontaneous elimination of PABC to release cytotoxin MMAE in its native form.

Non-specific uptake plays a crucial part in the distribution of ADCs into healthy cells that cause off-target side effects. The hydrophobicity of ADCs may contribute to their non-specific uptake by normal cells.^54^ Unlike the native mAb, ADC usually demonstrates a higher hydrophobicity because of the conjugation with the hydrophobic payload and the hydrophobic drug-linker combinations.^55^ Conjugation of hydrophobic payloads to ADCs can increase their surface hydrophobicity and internalization property, which facilitates the non-specific uptake into healthy cells without the target antigen, potentially leading to off-target effects and unintended toxicity in normal tissues.^54^ Moreover, the improvement of hydrophobicity may lead to aggregation of ADCs that enhance immunogenicity through activation of immune cells via Fcγ receptors (FcγRs).^56^ In addition, the non-specific endocytosis can also give rise to the off-site uptake of intact ADCs into healthy cells.^54^ The process of endocytosis is crucial in nerve cells for exchanging macromolecules or clusters of certain substances with the extracellular environment,^57^ and it is plausible that intact ADCs are internalized by nerve cells through this process. Apart from the non-specific uptake of intact ADC, the bystander effect and premature linker cleavage can cause the release of free MMAE into healthy cells that induce off-target toxicity.^58^ In all five approved MMAE-based ADCs, the mc-vc-PABC cleavable linker is employed for the conjugation between payload and antibody, which comprised of a thiol-reactive maleimidocaproyl (mc) group, a protease-sensitive valine-citrulline (vc) dipeptide, and a para-amino benzyloxycarbonyl (PABC) spacer (Figure 3).^1^ Vedotin, the mc-vc-PABC-MMAE drug/linker complex, undergoes the protease cleavage of valine-citrulline dipeptide linker by cathepsin B followed spontaneous elimination of PABC to release cytotoxin MMAE in its native form. Cathepsin B is a lysosomal protease with predominant expression in mammalian lysosomes and it is commonly found to be overexpressed in various types of cancer cells,^59^ therefore, it enables the precise and effective release of the MMAE payload in the targeted cancer cells. The design of “mc-vc-PABC-MMAE” linker-drug combination not only ensure the recognition of vc group by cathepsin B and can release the free MMAE as the product to diffuse into neighboring antigen-negative cells, achieving the bystander effect.^60^ Although ADCs incorporating “mc-vc-PABC-MMAE” linker-drug combination show greater systemic stability in physiological conditions, several serine protease (such as carboxylesterase 1C in mouse, neutrophil elastase in human) are known to cleave the “mc-vc-PABC” linker in circulation, resulting in the premature release of hypertoxic free MMAE to induce undesired off-target toxicity.^61^^,^^62^^,^^63^^,^^64^^,^^65^

The mechanisms for microtubule-targeting drug-induced peripheral neuropathy have been proposed to be related to the damage to the axon terminal, DRG neuron cells, and Schwann cells.^66^ The axonal transport is driven by molecular motors that run on the “tracks” formed by microtubules, which provide abundant drug target sites for microtubule-targeting drugs.^67^^,^^68^ Once the MMAE is taken up in the peripheral nerve cells through the direct method (free MMAE) or the indirect method (MMAE-based ADC uptake to release MMAE), MMAE binds to abundant binding sites along the length of microtubules and disrupts the microtubules network, resulting in the onset of peripheral neuropathy.^52^^,^^69^ Because of the relatively long length of peripheral nerves and the existence of blood-brain barriers, sensory peripheral neurons are more susceptible than central neurons to microtubule-targeting drug-induced damage.^70^^,^^71^^,^^72^ Moreover, the fenestrated structures in the blood-nerve barrier enable a higher exposure of sensory peripheral nerves to the microtubule-targeting drugs.^73^^,^^74^ Thus, microtubule-targeting drug-induced peripheral neuropathy is an atrophic effect starting at distal nerve endings, followed by changes in Schwann cell, the DRG neuronal cell body, or axonal transport.

In addition, recent studies have provided increasing evidence of the contribution of macrophages to the development of peripheral neuropathic pain.^75^^,^^76^^,^^77^^,^^78^ Specifically, in cases where nerve injury is caused by microtubule-targeting drugs, Schwann cells become activated and produce damage-associated molecular patterns (DAMPs), which in turn attract circulating leukocytes, such as M1-like macrophages, to the site of injury.^79^^,^^80^ In normal circumstances, tissue-resident macrophages are predominantly of the M2-like subtype, and they serve to maintain tissue homeostasis and protect the peripheral nervous system from damage.^81^ However, a shift in the macrophage population toward M1 macrophages promotes the release of various pro-inflammatory cytokines, including IL-1β, TNFα, CXCL1, CXCL12, *etc*. These cytokines trigger neuroinflammation and convey nociceptive information to DRG neurons, leading to the perception of pain (Figure 2).^82^^,^^83^^,^^84^

Although additional confirmation is needed, these findings underscore the biological intricacy underlying the induction of peripheral neuropathy by MMAE-based ADCs, highlighting the imperative need to utilize innovative approaches to elucidate this type of toxicity.

## Clinical diagnosis

Peripheral neuropathy is a complex disorder characterized by sensory, motor, and autonomic dysfunction.^85^ Sensory symptoms, particularly pain, are the most common and typically present as partial paresthesia in the feet and hands, including numbness, tingling, impaired vibration sensation, and changes in touch perception.^86^^,^^87^ Additionally, spontaneous burning, radiation, shock pain, mechanical/thermal dysalgesia, or hyperalgesia may be experienced.^88^ Conversely, motor symptoms are relatively infrequent and milder, presenting as mild weakness in the lower limbs and reduced or absent ankle reflexes.^13^^,^^89^ Furthermore, altered proprioception may occur, increasing the risk of falls or accidents.^90^ Abnormal autonomic symptoms are rare, with orthostatic hypotension, constipation, sexual dysfunction, and difficulty urinating being the most common.^91^

Additionally, peripheral neuropathy induced by MMAE-based ADCs has several unique features that differentiate it from other neuropathies.^10^^,^^52^^,^^92^ These features include (1) predominant sensory neuropathy characterized by pain, paresthesia, numbness, hypoesthesia, and hyperesthesia that may be accompanied by motor neuropathy in certain circumstances; (2) a distal, length-dependent “stocking-glove” distribution of peripheral neuropathy; (3) symmetric distribution of peripheral neuropathy lesions; (4) onset of peripheral neuropathy after administration of MMAE-based ADCs, which may be progressive and rapid; and (5) peripheral neuropathy severity increasing with dose and duration until the cessation of treatment. Identifying these diagnostic features can assist physicians in distinguishing MMAE-based ADC-induced peripheral neuropathy from other forms of neuropathy, making more accurate diagnosis and effective treatment.

## Risk factors

Peripheral neuropathy represents a known cumulative adverse event associated with MMAE-based ADCs.^93^^,^^94^ Although no expert consensus has been reached, the extent and duration of MMAE exposure within peripheral nerve tissue is considered the primary determinant governing peripheral neuropathy onset.^10^ For instance, in the phase I trials of brentuximab vedotin, the greater incidence and shorter onset time to peripheral neuropathy were observed in patients with weekly regimens compared to those receiving every three weeks.^36^^,^^95^ Furthermore, a higher body weight has been identified as an additional risk factor for peripheral neuropathy event when accounting for conjugate exposure.^69^ The increased risk associated with high body weight may relate to increased axonal nerve fiber length and surface area available for ADC exposure rather than obesity-induced inflammation or diabetes.^96^ There also appears to be a trend between elevated serum albumin concentrations and high peripheral neuropathy risk, potentially due to maleimide exchange enabling albumin-MMAE conjugate formation.^97^ Moreover, preliminary evidence suggests preexisting neuropathy, whether hereditary or acquired, may predispose individuals to exacerbated peripheral neuropathy upon MMAE-ADCs treatment.^98^^,^^99^ Patients with prior peripheral nerve damage likely exhibit enhanced sensitivity to additional ADC exposure, facilitating manifestation and progression of peripheral neuropathy symptoms.

## Management

In cases where there is a suspicion or confirmation of peripheral neuropathy induced by MMAE-based ADCs, primary management strategies may include withholding the dose, reducing the dosage, or permanently discontinuing the use of ADC drugs according to the severity of the peripheral neuropathy as graded by the CTCAE criteria. Typically, patients with grade 1 peripheral neuropathy during treatment with MMAE-based ADC drugs do not require dosage adjustments.^100^^,^^101^ However, in the case of grade 2 or 3 peripheral neuropathy, clinical judgment may be employed to determine whether treatment should be withheld until the neuropathy recovers to grade 1 or lower and the dosage should be reduced in subsequent treatments. For patients experiencing worsening peripheral neuropathy (grade 4), the ADC drugs should be permanently discontinued. However, permanent discontinuation is recommended for patients with grade 3 peripheral neuropathy treated with enfortumab vedotin and tisotumab vedotin.^102^^,^^103^

It is expected that a similar approach could be adopted for the other novel ADCs containing MMAE, whereby severe cases will require permanent discontinuation, while re-challenge will only be permitted for mild cases that have completely resolved. Nonetheless, ascertaining whether this approach is justifiable in all instances is still under investigation. A more in-depth understanding of this type of adverse event is vital, as it may facilitate the development of safe manage strategies. This may also involve the improvement of more objective peripheral neuropathy assessment tools and alternative techniques.

### Medical treatment

Patients undergoing antitumor therapy tend to prioritize the therapeutic effect over peripheral neuropathy. In order to avoid the interference of anticancer efficacy, dose delay, dose reduction, or dose discontinuation is currently recommended in response to peripheral neuropathy when patients are receiving antitumor therapy. However, if a severe peripheral neuropathy was observed in patients who have completed anticancer therapy, some medications could be implemented to enhance patients' treatment compliance and improve patients' quality of life and prognosis. For example, some studies have suggested that duloxetine, acetyl-*l*-carnitine, amifostine, and methylcobalamin may have beneficial effects in relieving symptoms of peripheral neuropathy.^104^^,^^105^^,^^106^^,^^107^

(1) Duloxetine: Duloxetine is a novel selective serotonin and norepinephrine reuptake inhibitor with proven efficacy and tolerability in treating various types of chronic pain.^108^ It has been recommended as a first-line drug by the International Pain Society for the treatment of neuropathic pain. Docetaxel was shown to induce mechanical and thermal dyspepsia, as well as abnormal cold dyspepsia, but continuous oral administration of duloxetine was effective in reducing the resulting neuropathic pain symptoms.^109^ The American Society of Clinical Oncology (ASCO) guidelines suggest that duloxetine has the strongest data support for the treatment of neuropathic pain related to peripheral neuropathy.^110^ In a multicenter, randomized, double-blind, and crossover trial, patients receiving duloxetine initially had significantly lower pain scores than those receiving a placebo.^104^ A recent prospective study in China confirmed that duloxetine effectively prevents peripheral neuropathy (OR = 5.426; 95% CI: 1.898–15.514; P = 0.002).^111^ Currently, the FDA has approved duloxetine for the treatment of diabetic peripheral neuropathy and fibromyalgia. Given the effect of duloxetine, it is reasonable to assume that duloxetine may be a promising agent to reduce peripheral neuropathy symptoms caused by MMAE-ADCs.
(2) Acetyl-L-carnitine (ALC): ALC is a trimethylated amino acid that plays a crucial role in intermediate metabolism and has demonstrated high efficacy and tolerance in the treatment of various peripheral neuropathies.^112^^,^^113^ It was shown that ALC could prevent peripheral neuropathy symptoms caused by paclitaxel and promote the recovery of nerve conduction velocity and up-regulation of metabolic glutamate receptors in the dorsal root ganglia to induce analgesia in peripheral neuropathy animal models.^114^ However, recent studies have suggested that ALC may worsen neuropathic symptoms. Hershman et al. conducted clinical observations on breast cancer patients receiving paclitaxel chemotherapy and compared the efficacy of ALC with placebo in treating peripheral neuropathy.^115^ Their findings revealed that ALC, compared to the placebo, led to worsening peripheral neuropathy symptoms.^115^ This result persisted even after two years of discontinuing ALC, indicating that the clinical application of ALC should be approached with caution.^116^
(3) Amifostin: Amifostin is a normal cell protectant that can be used as an adjunct therapy for tumor radiotherapy or chemotherapy.^117^ Studies suggested that amifostine has a preventive effect on neurotoxicity caused by platinum-based chemotherapy drugs, protecting peripheral nerves without reducing the efficacy of the chemotherapy drugs.^118^ In a randomized trial by Wang Fen et al., 86 patients with gastrointestinal tumors receiving FOLFOX6 chemotherapy were divided into amifostine and control groups.^119^ After eight cycles of chemotherapy, the incidence of grade 3 or 4 neurotoxicity was significantly lower in the amifostine group than in the control group. Additionally, the sensory nerve conduction velocity of the median and peroneal nerves was significantly better in the amifostine group compared to the control group. However, the two groups had no significant difference in the short-term response rate of chemotherapy.
(4) Mecobalamin: also known as methyl vitamin-B12. As the active form of vitamin B12, mecobalamin is easier to enter neuron cells because of the high affinity with nerve cells, which can promote the utilization of folic acid and nucleic acid metabolism.^107^ Mecobalamin has been demonstrated to promote nucleic acid and protein synthesis in nerve cells, facilitating the regeneration of nerve myelin sheaths and axons.^120^ Moreover, it could repair the damaged nerve cells and improve nerve conduction velocity.^121^

### Non-medical treatment

In addition, some non-medical treatments were adopted to mitigate peripheral neuropathy to some extent. As an auxiliary method, acupuncture and moxibustion are safe and effective with a low incidence of adverse reactions. In a randomized controlled trial to evaluate the feasibility and safety of acupuncture intervention in patients with moderate to severe peripheral neuropathy with breast cancer, eight weeks of acupuncture intervention compared with conventional care significantly improved peripheral neuropathy neurosensory symptoms in patients with mild and moderate breast cancer, with no serious adverse effects observed.^122^ However, future clinical design, including appropriate sample size, adequate follow-up time, and clear study endpoints, is needed to establish a peripheral neuropathy gold standard acupuncture regimen. Moreover, cryotherapy and compression therapy have been reported as effective treatments for paclitaxel-induced peripheral neuropathy with generally well-tolerated and safe.^123^ As demonstrated in clinical trials, the use of surgical gloves for compression therapy has shown promise as a potentially safe and effective treatment option.^124^ Additionally, the injection of anesthetics or physical stimulation around the nerve trunk for nerve blocker could be used for accurate positioning and relatively few adverse reactions, including paraspinal nerve block, selective nerve root block, sympathetic nerve block, and other treatment methods, which are widely used in the treatment of peripheral neuralgia.^125^

## Diagnostic flowchart

**Figure 4:**
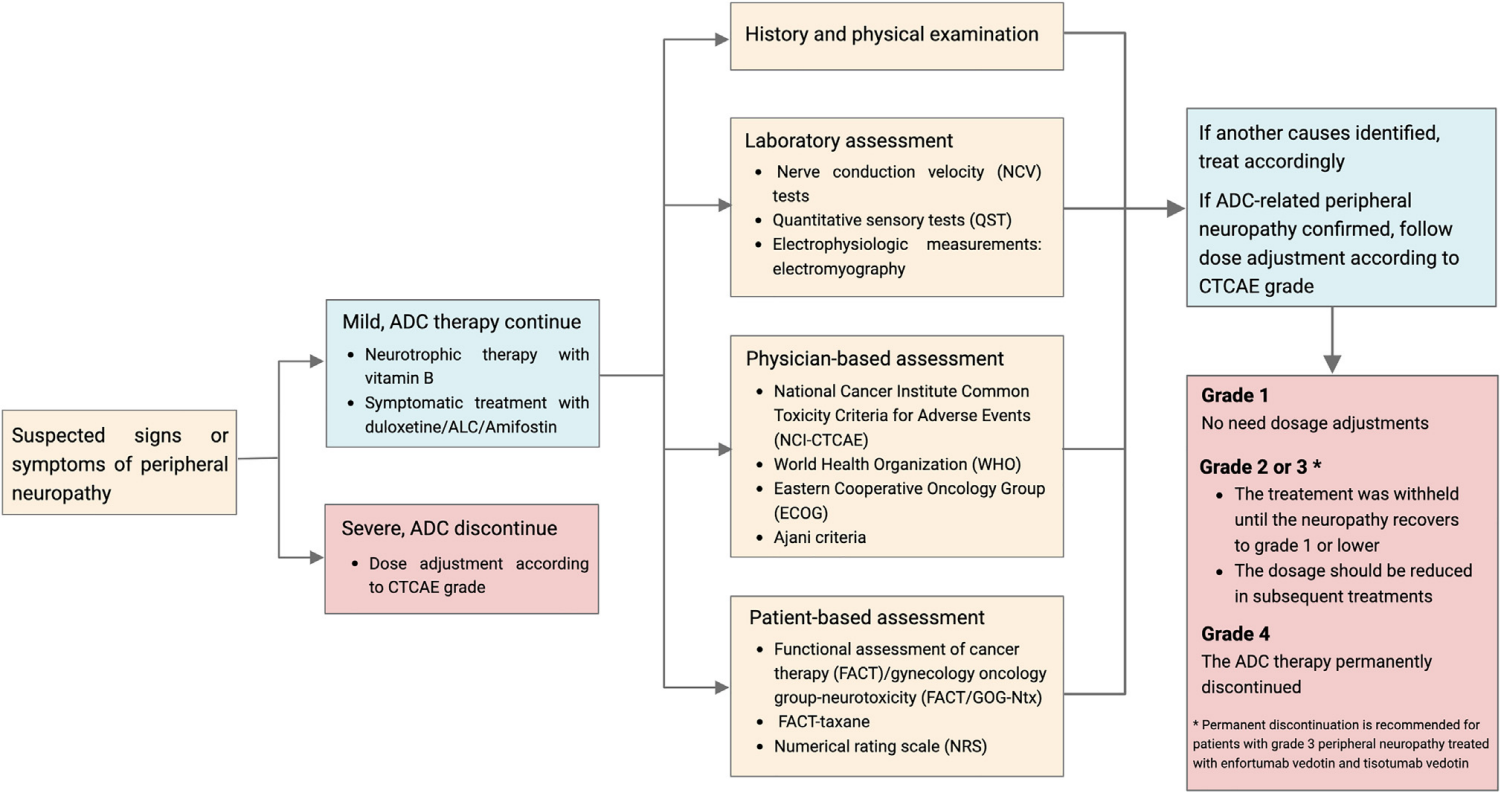
Diagnostic flowchart for peripheral neuropathy associated with MMAE-based antibody-drug conjugates (ADCs)

Drawing upon our current knowledge and understanding, we proffer algorithmic recommendations for the diagnosis and management of peripheral neuropathy associated with MMAE-based ADCs, as depicted in Figure 4. If suspected signs or symptoms of peripheral neuropathy appear, except for severe cases that need to discontinue ADC immediately, we recommend adopting the neurotrophic therapy with B vitamins or symptomatic treatment with duloxetine/ALC/amifostin to relieve discomfort. Subsequently, a comprehensive medical history and physical examination should be performed to rule out alternative causes of peripheral neuropathy symptoms. It is advisable to perform diagnostic tests, including routine objective laboratory, physician-based, and patient-based assessments. When peripheral neuropathy was confirmed to be associated with ADC treatments, the dose adjustment was carried out according to the CTCAE grade.

## Future perspectives

Currently, the number of survivors for cancer patients is increasing due to the rapid development of novel anticancer therapies. Although the mechanism of antitumor action of chemotherapy drugs is clear, the mechanism leading to peripheral neuropathy may differ, leading to the unpredictability of peripheral neuropathy occurrence and development. The occurrence, severity, and duration of symptoms vary widely among individuals. To achieve better, comprehensive, and satisfactory efficacy, further in-depth and extensive exploration is necessary, and there is still a long path ahead in this area of research.

One especially attractive route might be the improvement of ADC technology to reduce the incidence and severity of peripheral neuropathy associated with ADC therapy. Traditional ADCs often utilize random conjugation methods, which can result in heterogeneous mixtures with varying drug-to-antibody ratios (DAR). However, site-specific conjugation techniques, such as enzymatic conjugation or site-specific bioconjugation, were recently developed to allow for precise and controlled drug attachment to specific sites on the antibody.^126^ It can enhance ADC stability, improve pharmacokinetics, and reduce off-target toxicity, potentially minimizing peripheral neuropathy. For instance, Matsuda et al. have developed a site-specific chemical conjugation method using IgG Fc-afinity reagents.^127^ It could significantly expand the therapeutic index of ADC compared to stochastic conjugation methods. Through site-specific conjugation technology, Pavel Strop et al. generate several highly loaded ADCs that exhibited good safety profiles with higher exposure in comparison to the conventional conjugates.^128^ Moreover, payload diversification is expected to play a key role for reducing the risk of peripheral neuropathy. Since MMAE is the main driver for ADC-induced peripheral neuropathy, the development of novel and potent cytotoxins (such as pyrrolobenzodiazepine monomers/dimers, indolino-benzodiazepines or cyclopropabenzindolone monomers/dimers) as the ADC payloads would be effective to mitigate the side effects of peripheral neuropathy.^129^ Furthermore, the advanced linker technologies, such as the non-peptide linkers using the bioorthogonal click chemistry, enable payload release in the target cell’s intracellular compartments only.^130^^,^^131^ The controlled payload release can reduce exposure to normal tissues, including peripheral nerves, thereby reducing the risk of peripheral neuropathy. These advances in ADC technology hold promise for mitigating peripheral neuropathy. However, it’s important to note that these advancements are still in various pre-clinical stages of development and evaluation.^132^ Clinical trials and further research are needed to validate their efficacy, safety, and impact on peripheral neuropathy. Collaborative efforts between researchers, clinicians, and pharmaceutical companies are vital for advancing ADC technology and improving the therapeutic profile of ADCs to minimize neurotoxicity, including peripheral neuropathy.

Secondly, early detection and prevention of peripheral neuropathy associated with MMAE-based ADC therapy can help mitigate its impact and improve patient outcomes. To this end, a comprehensive, convenient, and effective assessment tool is urgently needed to reflect the actual level of patients to the greatest extent. Peripheral neuropathy can cause peripheral nerve damage in patients, reducing their ability to perform daily activities and their quality of life. And peripheral neuropathy symptoms also affect the patient’s psychological, social, and spiritual aspects. Therefore, an ideal comprehensive assessment tool for peripheral neuropathy should include an assessment of the patient’s symptoms and their impact on quality of life and psychological, spiritual, and social relationships.

Additionally, better medical treatments will be required to improve peripheral neuropathy outcomes. The application of nanotechnology-based drug delivery systems presents a promising approach to overcome the low solubility and the bioavailability of certain compounds to relieve peripheral neuropathy. The utilization of nanocarriers in pain management represents a nascent and burgeoning field of inquiry with substantial prospects for clinical advancement.^133^^,^^134^

## Conclusions

In conclusion, monitoring and managing peripheral neuropathy in patients receiving MMAE-based ADCs are essential for promoting patient well-being, maintaining treatment efficacy, and optimizing treatment outcomes. A proactive and multidisciplinary approach involving oncologists, neurologists, pain specialists, and supportive care teams is crucial to effectively monitor, assess, and manage peripheral neuropathy in these patients. We expect the establishment of better guidelines for the management and monitoring of peripheral neuropathy induced by MMAE-based ADC in the near future.
